# A unique strain of community-acquired *Clostridium difficile* in severe complicated infection and death of a young adult

**DOI:** 10.1186/1471-2334-13-299

**Published:** 2013-07-01

**Authors:** Orville D Heslop, Karen Roye-Green, Kathleen Coard, Michael R Mulvey

**Affiliations:** 1 Department of Microbiology, University of the West Indies, Kingston, Jamaica; 2 Department of Pathology, University of the West Indies, Kingston, Jamaica; 3 National Microbiology Laboratory, 1015 Arlington Street, Winnipeg MB R3E 3R2, Canada

**Keywords:** *Clostridium difficile*, *Klebsiella pneumoniae*, Community-Acquired Infection, Diarrhoea, Clindamycin, Pseudomembranous Colitis, Toxic Megacolon

## Abstract

**Background:**

*Clostridium difficile* is the major cause of nosocomial antibiotic-associated diarrhoea with the potential risk of progressing to severe clinical outcomes including death. It is not unusual for *Clostridium difficile infection* to progress to complications of toxic megacolon, bowel perforation and even Gram-negative sepsis following pathological changes in the intestinal mucosa. These complications are however less commonly seen in community-acquired *Clostridium difficile* infection than in hospital-acquired *Clostridium difficile* infection. To the best of our knowledge, this was the first case of community-acquired *Clostridium difficile* infection of its type seen in Jamaica.

**Case presentation:**

We report a case of a 22-year-old female university student who was admitted to the University Hospital of the West Indies, Jamaica with a presumptive diagnosis of pseudomembranous colitis PMC. She presented with a 5-day history of diarrhoea following clindamycin treatment for coverage of a tooth extraction due to a dental abscess. Her clinical condition deteriorated and progressed from diarrhoea to toxic megacolon, bowel perforation and Gram-negative sepsis. *Clostridium difficile* NAP12/ribotype 087 was isolated from her stool while blood cultures grew *Klebsiella pneumoniae*. Despite initial treatment intervention with empiric therapy of metronidazole and antibiotic clearance of *Klebsiella pneumoniae* from the blood, the patient died within 10 days of hospital admission.

**Conclusions:**

We believe that clindamycin used for coverage of a dental abscess was an independent risk factor that initiated the disruption of the bowel micro-flora, resulting in overgrowth of *Clostridium difficile* NAP12/ribotype 087. This uncommon strain, which is the same ribotype (087) as ATCC 43255, was apparently responsible for the increased severity of the infection and death following toxic megacolon, bowel perforation and pseudomembranous colitis involving the entire large bowel. *K. pneumoniae* sepsis, resolved by antibiotic therapy was secondary to *Clostridium difficile* infection. The case registers community-acquired *Clostridium difficile* infection as producing serious complications similar to hospital-acquired *Clostridium difficile* infection and should be treated with the requisite importance.

## Background

*Clostridium difficile* (*C*. *difficile*) is the major cause of nosocomial antibiotic-associated diarrhoea [1,2], while secondary Gram-negative shock in community-acquired *Clostridium difficile* infection (CA-CDI) may occur following bowel perforation in hospitalised patients [3]. CA-CDI is defined as symptoms that occur in the community or within 48 hours of admission to a hospital, provided symptoms from onset were > 12 weeks after the last discharge from a hospital [3]. CA-CDI occurs less frequently than hospital-acquired *C. difficile* infection (HA-CDI). It is usually less severe and presents more frequently among females with a median age of 50 years compared to 72 years with HA-CDI [3]. The most common risk factors associated with CDI are broad-spectrum antibiotics including third generation cephalosporins, clindamycin, penicillins and, more recently, fluoroquinolones [2,4]. However, virtually all antibiotics are potential risk factors for CDI [5]. We highlight the unusual strain *C. difficile* ribotype 087 and pulsed-field gel electrophoresis (PFGE) type 0515 associated with severe complicated CDI, which was being seen for the first time in Jamaica. *Klebsiella pneumoniae* (*K. pneumonia)* sepsis, a subsequent clinical outcome, was diagnosed from positive blood cultures after a 5-day history of apparent clindamycin-induced diarrhoea in CA-CDI.

## Case presentation

A 22-year-old female presented to the University Hospital of the West Indies, Jamaica for further investigation and management following transfer from a local Government hospital where she presented with a 5-day history of diarrhoea and fever. The diarrhoea commenced 5 days after starting clindamycin therapy for a recent tooth extraction due to a dental abscess. Despite discontinuation of clindamycin therapy and the introduction of chloramphenicol and metronidazole empiric therapy, the diarrhoea and fever continued.

Physical examination of the patient revealed a febrile female in severe cardiopulmonary distress with tachycardia, HR beats/150 min, and blood pressure 150/100 mm/Hg. Laboratory investigations showed haemoglobin of 10.4 gm/dl, white cell count of 2.1 × 10^9^/litre, and platelet count of 182 × 10^9^/litre. Diarrhoea persisted and progressed to toxic megacolon and bowel perforation. Blood cultures (brain heart infusion and thioglycollate broths) collected on admission grew *K. pneumoniae* from all four bottles after 24 hours incubation at 37°C. Diarrhoeal stool specimen sent the day after admission was positive for *C. difficile* toxin A/B (ELISA; Alexon Inc. 1190 Borregas Ave., Sunnyvale, CA. 94089–1302) and the corresponding organism isolated on selective culture medium, cycloserine, cefoxitin, fructose agar (CCFA).

In the interim period of empiric therapy, susceptibility testing on *K. pneumoniae* by the Kirby Bauer disc diffusion method showed susceptibilities to ceftriaxone, co-trimoxazole, ceporin, amikacin, gentamicin, ceftazidine, and augmentin, as well as resistance to chloramphenical, piperacillin and ampicillin. The patient continued therapy on intravenous metronidazole and with the introduction of ceftriaxone and gentamycin she became afebrile with subsequent blood cultures becoming sterile. Hypotension, pulmonary oedema, leucopenia, and thrombocytopenia persisted despite appropriate therapy and intensive care management. The patient died 10 days after hospital admission and an autopsy was performed. The most significant finding of the autopsy was multiple discrete plaques of yellowish exudate on the mucosal surface of the entire large bowel typical of pseudomembranous colitis (PMC), which was florid. Another noteworthy pathologic finding was that of markedly overweight lungs with features in keeping with adult respiratory distress syndrome.

**Figure 1:**
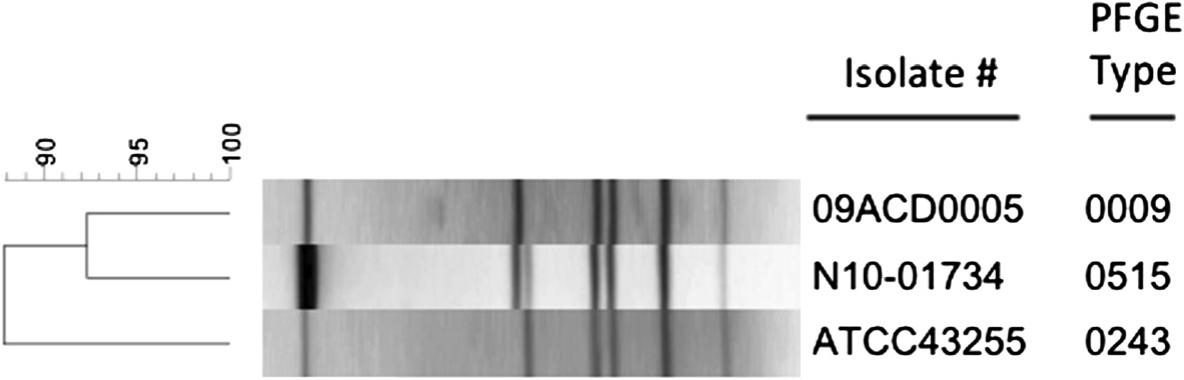
**Dendrogram depicting the *Sma*l *C. difficile *fingerprint patterns from isolates used in this study; N10-01734 (clinical isolate); 09ACD0005 (typical NAP12 isolate); ATCC43255 (reference strain).** The dendrogram was generated using BioNumerics v 3.5 (Applied Maths, Belgium) using a band tolerance of 1.0%.

Polymerase chain reaction (PCR) was used to confirm the presence of *C. difficile* triphosphate isomerase (*tpi*), toxin genes *tcdA* and *tcdB*, the lack of binary toxin *cdtB* gene, and no deletion in *tcdC* gene [6]*.* PCR ribotyping on the isolate revealed a ribotype of 087 and the pulsed-field gel electrophoresis (PFGE) macrorestriction pattern identified a fingerprint type 0515, not previously seen using this method, and when compared to a national collection of over 700 unique fingerprint types from more than 7,100 CDI isolates (Figure 1). However, the fingerprint pattern was closely related to the North American Pulsotype (NAP) NAP12 strain with only a one band difference (Figure 1). The minimal inhibitory concentrations (MIC) determined by Etest were as follows: susceptible to metronidazole 0.094 ug/ml; vancomycin 1 ug/ml; moxifloxacin 1 ug/ml; rifampicin < 0.002 ug/ml; tigecycline 0. 125 ug/ml; and resistant to clindamycin 256 ug/ml. *K. pneumoniae* was isolated from 2 sets of blood cultures following overnight incubation at 37°C.

The severe clinical outcomes seen in the present case were not unusual though more commonly seen in HA-CDI [3]. In addition to these clinical presentations, associated hypotension and admission to the intensive care unit classified this patient as a severe complicated case of CDI [7]. In a large study covering the period 1991–2005 in Olmsted County, Minnesota, it was interesting to note that only 4% of CA-CDI had progressed to severe complicated CDI [8]. It was further noted that such cases are usually associated with a significantly older age group with a median age of 80 years [8]. The manifested clinical involvements along with a significant age difference are important features to consider in CA-CDI and should serve to alert clinicians that there is always a potential risk of young adults progressing to severe complicated CDI. The source of *K. pneumoniae* sepsis in the patient was presumably the gastrointestinal tract, as there were no symptoms or signs to suggest other systems involvement. Notwithstanding treatment with metronidazole, the patient was unresponsive and progressed to toxic megacolon and bowel perforation. These were probably due to the failure to administer appropriate and adequate clinical and surgical interventions.

Oral metronidazole remained the preferred first line drug for treatment while vancomycin is reserved for severely ill patients and recurrence of *C. difficile* colitis [9]. Interestingly, these interventions including the use of adjunctive intracolonic vancomycin therapy and total colectomy, as recommended options, were not followed in the management of the present case. It is important to note that PMC was confirmed by autopsy. The failure to apply the optimal required clinical and surgical interventions during the patient’s hospitalisation were probably due to the rapid progression of these clinical outcomes.

With the exception of toxins A and B produced by this isolate, there was no deletion observed in the negative regulator *tcdC* gene suggesting normal toxin expression. However, ATCC 43255, which also has a wild type *tcdC*, has shown increased toxin expression [10]. Importantly, ATCC 43255 has the same ribotype (087) as the clinical isolate and only a single band difference in the DNA fingerprints was observed between the two isolates (Figure 1). Based on the similarity of ribotypes and fingerprint patterns between the two isolates, one could speculate that the rapid progression to PMC in the patient may be due to increased toxin expression. Ribotype 087 is the predominant strain in Hungary but is uncommon internationally [11].

The patient had no history of hospitalisation 12 months prior to infection and no health care-associated risk factors to CDI were noted. On the contrary, this young adult female was actively pursuing a university education, which was disrupted by CA-CDI [3]. The progression to toxic megacolon in the patient was not a predicted clinical outcome even after hospitalisation. The presence of a perforated colon was a clear indication for surgical intervention, especially if there were unresponsiveness to other treatments and if clinical improvement was not noted within 2 to 3 days of patient management [12].

## Conclusions

To the best of our knowledge, this was the first case of CA-CDI of its type seen in Jamaica, and was fraught with many challenges including diagnosis and clinical management. The clinical manifestations of CA-CDI rapidly progressed to serious complications but were not given the appropriate and timely treatment interventions. We believe that clindamycin used for coverage of a dental abscess was an independent risk factor that initiated the disruption of the bowel micro-flora resulting in overgrowth of NAP12/ribotype 087. This was apparently responsible for the increased severity of CDI seen in the patient. The progression to death was the result of complications from florid PMC while *K. pneumoniae* sepsis was secondary to bowel perforation initiated by pathological changes in the intestinal mucosa.

These findings underscore two important points that clinicians should note: [1] the need to use clindamycin more discriminately in spite of its excellent coverage as a broad-spectrum antibiotic, and [2] to be more alert in recognising the potential risk of CA-CDI progressing to severe complicated CDI in young adults. Additionally, clinicians and other healthcare providers should be prudent to employ appropriate patient management to reduce the morbidity and mortality rate among hospitalised *C. difficile* infected individuals.

## Consent

Written informed consent was obtained from the parents of the patient for publication of this Case report and any accompanying images. A copy of the written consent is available for review from the Series Editor of this journal.

The investigation of *C. difficile* infection whether acquired in a hospital or in the community is an ongoing research study, approved by the UHWI Ethics Committee. The present case study would be included even though this particular case occurred sometime before we received ethical approval. Please see attached letter from the UHWI Ethics Committee.

## Competing interests

All authors declare that they have no competing interests.

## Authors’ contributions

All authors read and approved the final manuscript. OH wrote the manuscript and conducted the initial laboratory diagnosis of the present case. KRG wrote the case report along with KC who wrote the pathological findings. MM conducted the genotyping of the clinical isolate, and contributed to the molecular analysis and to writing the manuscript.

## Pre-publication history

The pre-publication history for this paper can be accessed here:

http://www.biomedcentral.com/1471-2334/13/299/prepub
